# Culturally responsive research ethics: How the socio-ethical norms of *Arr-nar*/*Kreng-jai* inform research participation at the Thai-Myanmar border

**DOI:** 10.1371/journal.pgph.0001875

**Published:** 2023-05-04

**Authors:** Napat Khirikoekkong, Supa-at Asarath, Suphak Nosten, Borimas Hanboonkunupakarn, Nattapat Jatupornpimol, Jennifer Roest, Michael Parker, Francois Nosten, Rose McGready, Phaik Yeong Cheah, Maureen Kelley

**Affiliations:** 1 Shoklo Malaria Research Unit, Faculty of Tropical Medicine, Mahidol University, Mae Sot, Thailand; 2 Mahidol Oxford Tropical Medicine Research Unit, Faculty of Tropical Medicine, Mahidol University, Bangkok, Thailand; 3 Department of Clinical Tropical Medicine, Faculty of Tropical Medicine, Mahidol University, Bangkok, Thailand; 4 Wellcome Centre for Ethics & Humanities, Nuffield Department of Population Health, University of Oxford, Oxford, United Kingdom; 5 The Ethox Centre, Nuffield Department of Population Health, University of Oxford, Oxford, United Kingdom; 6 Centre for Tropical Medicine & Global Health, Nuffield Department of Medicine, University of Oxford, Oxford, United Kingdom; ESIC Medical College & PGIMSR, INDIA

## Abstract

Despite advances, international research ethics guidelines still tend to consist of high-level ethical principles reflecting residual influence from North American and European traditions of ethics. Local ethics committees and community advisory boards can offer more culturally-sensitive approaches to training but most institutions lack substantive practical ethics guidance to engage rich moral understandings in day-to-day research practice in diverse cultural contexts. To address this gap, we conducted an international series of qualitative research ethics case studies, linked prospectively to active research programs in diverse settings. Here, we share findings from two case studies with a research team working on malaria and hepatitis B prevention with pregnant women in clinics serving migrants along the Thai-Myanmar border. In this sociocultural ethical analysis, we consider how core ethical requirements of voluntary participation, provision of fair benefits, and understandings of research risks and burdens are shaped, enriched, and in some instances challenged, by deep-seated and widespread Burmese, Karen and Thai cultural norms known as *Arr-nar* (in Burmese and Karen) or *Kreng-jai* (in Thai), encompassing multiple meanings including consideration for others and graciousness. We offer a model illustrating how one might map ethically significant sociocultural influences across the research practice pathway and close with lessons for developing a more culturally responsive research ethics practice in other international settings.

## Introduction

International research is governed by a complex combination of country-level research ethics guidelines, international ethics guidelines, and regional or community ethical oversight through community advisory boards. Research ethics guidance at the institutional level, informing much of practice and training, still tends to consist of fairly high-level ethical principles and these principles continue to reflect a strong, residual influence from North American and European traditions of ethics [1–3]. Such principles often lack sufficient depth to engage rich moral understandings in day-to-day research practice in diverse cultural contexts and may even be in tension with local moral norms [4]. Ethical concepts central to international guidance—such as ‘vulnerability’ and ‘autonomy’—sometimes lack direct translations and the meanings may fail to resonate within local norms and practice [5]. Emerging scholarship attempts to contextualize international research ethics across diverse contexts, using ‘empirical ethics’ and social science methods to explore issues such as ancillary care obligations, capacity and consent, vulnerability and agency, fair benefits, and participant and community engagement within local sociocultural contexts [4, 6–13].

Building on these foundations in empirical research ethics, we conducted the Resilience, Empowerment and Advocacy in Women’s and Children’s Health research project (REACH). This five-year collaborative research ethics study took place in Thailand, Kenya and South Africa and aimed to understand, the lived-experience of research teams and participants involved in a range of global health research programs in women’s and children’s health. In this paper we focus on our study in Tak Province, Thailand, where we conducted a prospective embedded ethics study linked to two ongoing research studies with pregnant women that were undertaken in clinics serving highly marginalized populations of Burman, Karen and other ethnic groups residing on both sides of the Thai-Myanmar border (henceforth referred to as ‘border context’). Findings related to structural vulnerability and agency in research participation have been presented elsewhere [14].

Throughout our data, we discovered that research ethics issues in this border context could not be properly understood without engaging broader sociocultural-moral norms distinctive of this region—the Burmese, Karen and Thai norms surrounding *Arr-nar* or *Kreng-jai* [15]. We conducted a sociocultural ethical analysis of these ideas, exploring how central ethical considerations of voluntary participation, provision of fair benefits, and understandings of research risks or burdens are shaped, enriched, and in some instances challenged, by these regional norms.

## Methods

### Ethics statement

The REACH study was reviewed and approved by the Ethics Committee of the Faculty of Tropical Medicine, Mahidol University (MUMTH 2017-063-01), the Oxford Tropical Research Ethics Committee (OxTREC 529–17) and the Tak Province Community Ethics Advisory Board (T-CAB) (20170624/TCAB-07).

All REACH study participants had given their written informed consent to participate in this study, and were informed that data collected would be used for publication purposes. All participants in the PVM workshop had given their consent and signed media release forms for use of their drawings and videos for the purpose of academic publication and teaching.

### Setting and study site

Our study was conducted in Tak Province, North-western Thailand, in districts along the Thai border with Karen State, Myanmar. On either side of the border are mountainous regions with dirt roadways, heavy evergreen forests, and agricultural areas. Historical and persistent ethnic conflicts in this area affect everyone living and working in the region. Migrants residing along this border region can be classified into three groups: 1) ‘stable’, typically with documents and permission to work in Thailand, 2) ‘cross-border’, those without documents, typically living on the Myanmar side and crossing into Thailand for work or temporary living while working, and 3) ‘unstable’, those without documentation and without a safe, stable living situation in Thailand [14]. Those who are residing in refugee camp are provided with basic healthcare and protection, hence not considered to be migrants [16, 17]. Our study primarily focused on participants who undocumented ‘cross-border’ and ‘unstable’ migrants [14].

The study site was the Shoklo Malaria Research Unit (SMRU), Tak Province, Thailand, a field research unit of Mahidol-Oxford Tropical Medicine Research Unit (MORU), Thailand. For 35 years, SMRU has been providing humanitarian health services and conducting health research for the underserved population of migrant and cross-border communities, addressing local health challenges including malaria, tuberculosis, and maternal and child health [18, 19]. SMRU also has a comprehensive community engagement program that helps to ensure that health programs and research are relevant and responsive to community needs [20, 21]. SMRU staff include doctors, researchers, healthcare workers at its clinics and clinical technicians at its laboratories, most of whom are long-term residents of Thailand, from all parts of the world. Frontline healthcare workers and engagement staff are locally recruited and are of Karen and Burmese ethnicity.

### Study design, recruitment, and data collection

The REACH study was designed as an embedded empirical ethics study [22, 23]. The choice of site and boundaries of the case study were informed by our objective: to understand the ethical issues, experiences, and daily challenges of conducting research in the border region with its complex cultural, political, and economic challenges and marginalized migrant population [24, 25]. The case boundaries were determined by the research/health services institution, SMRU, situated within the historical, political, and economic context and demographics of the migrant populations in this border region. Within these boundaries, our primary qualitative data collection was linked to two approved clinical research studies at SMRU, which were 1. hepatitis B prevention during pregnancy or TDF [26], and 2. treatment of uncomplicated malaria during pregnancy or DMA [27], as shown in the Table 1 below.

**Table 1:** Key features and overview of linked case studies.

Study acronym: TDF Trials identifier: NCT02995005	Study acronym: DMA Trials identifier: NCT0154248
**Study objective:** To estimate the time to complete hepatitis B virus (HBV) DNA suppression in HBV DNA positive women who start tenofovir in the late first or early second trimester; and to estimate the proportion of women with HBV DNA at delivery.	**Study objective:** To determine the efficacy and safety of dihydroartemisinin-piperaquine, artesunate-mefloquine and artemether-lumefrantrine (augmented dose) for treatment of uncomplicated malaria in pregnant women.
**Study period:** May 2017 –December 2021	**Study period:** October 2009 –December 2018
**Study design:** Single arm, open label, tenofovir treatment intervention study.	**Study design:** Randomised controlled trial, 1:1:1, open label
**Phase of trial:** Phase IV	**Phase of trial:** Phase III
**Sample size:** 170 (actual)	**Sample size:** 511 (actual)
**Participants and criteria** • Pregnant women with estimated gestation of between 12 to 20 weeks. • Aged 16–45 years; and their offspring.	**Participant and criteria** • Women with acute uncomplicated malaria, and at 2^nd^ and 3^rd^ trimester of pregnancy. • Aged 18–45 years; and their offspring.
**Study procedures** • Monthly follow up from enrolment until infant is 2 months old. • Single visit when infant is 4 and 6 months old. • Venous blood draw monthly for safety (mother kidney and liver test) and HBV viral load in mother and single venous blood draw for baby (at 2 months of age).	**Study procedures00** • Up to 4 years of follow-up from enrolment in pregnancy (to infant age 4 years). • Daily follow-up until malaria smear is negative, then weekly to day 63, then every 1–2 weeks until delivery. • For infant: in year 1, monthly follow up, and 3 monthly visits thereafter. • Small volume finger prick samples (x10) for drug level analysis in first 42 days.
**Risks** • Risk of liver flare due to disease; risk is increased when the drug is ceased post-partum. Flare is usually biochemical without symptoms, but can be severe and is treatable.	**Risks** • All drugs used in the study are recommended by international malaria treatment guidelines. • The dose of artemether-lumefantrine is higher in the study than in non-pregnant women. There may be side effects related to the higher dose.
**Direct benefits, reimbursement, and compensation** • Study population unable to access Tenofovir outside of study and/or unaffordable—cost 1,300 baht (£ 30.17 GBP) per month. Additional cost for liver tests. This is equivalent to 11 days of daily wages earned. • Participant will be informed of their HBV status. • Compensated 50 baht (£ 1.15 GBP) per study visit • Reimbursed for transportation cost for study follow ups.	**Direct benefits, reimbursement, and compensation** • Participants will be treated for free with the same drugs. (dihydroartemisinin-piperaquine, artesunate-mefloquine) in the same clinics. • Compensated 100 baht (£ 2.30 GBP) per study visit. • Reimbursed for transportation cost for study follow ups.
**Potential benefits** Exploring pharmacokinetics is critical for maximizing the efficacy and minimizing side effects of any anti-viral interventions during pregnancy.	**Potential benefits** Findings and data from study will potentially contribute to treatment improvement on uncomplicated malaria in pregnant women.
**Sponsor; Funder** University of Oxford; Thrasher Research Fund (with core funding by the Wellcome Trust)	**Sponsor; Funder** University of Oxford; Holleykin Pharmaceuticals (with core funding by the Wellcome Trust)

We relied on purposive sampling to identify and recruit our participants (Table 2). We relied on a mix of in-depth interviews (IDIs) and focus group discussions (FGDs). Wherever possible, FGDs were carried out with pre-existing groups, for example, with the community advisory board members, healthcare workers from the same clinic, or researchers from the same unit. We conducted 32 IDIs and 10 FGDs with four groups of participants as shown in Table 2 [14]. All participants were 18 years of age and above, provided written consent in a language that they understood or provided verbal consent in the presence of a literate, impartial witness.

**Table 2:** REACH recruitment by group and type of data collection.

Data collection method	Participant Group	Female	Male
**In-depth Interview**	Group 1: Pregnant women who joined one of the linked studies and their family members	12	2
	Group 2: Research team members from the linked studies	6	2
	Group 3: Member of Tak Province Community Advisory Board (T-CAB)^a^ and member of local ethics committee	1	3
	Group 4: Participants with in-depth knowledge or experts on health-related topics on Thai-Myanmar border	1	5
**Total IDIs**		20	12
**Focus Group Discussion**	Group 1: Pregnant women who joined one of the linked studies and their family members	4 groups	
	Group 2: Research team members from the linked studies	4 groups	
	Group 3: Members of Tak Province Community Advisory Board (T-CAB)* and member of local ethics committee	2 groups	
**Total FGDs**		10 groups of FGDs (total 48 persons)	

* T-CAB is a community advisory board established in 2009 to advise researchers on practical and ethical aspects of research and health programmes on the Thai–Myanmar border [20]

Lead interviewers were independent of the linked clinical studies and are native Karen-Burmese speakers with a deep understanding of the research setting and the cultural context (NK, SN). Interviews with Thai and some international researchers and key informants were conducted by a native Thai speaker (NJ) and native English speaker (MK). Data collection ran from December 2017 to March 2019, until we achieved data saturation and no new themes identified.

Topic guides for IDIs and FGDs (see supporting information S1 and S2 Appendices) were drafted in English, then translated with much discussion to Karen, Burmese, and Thai. During creation of the guides, and iterative revisions, the team discussed subtleties of meaning in translation. For example, the concept of ‘vulnerability’ did not have a direct Karen or Burmese translation, so, the guide included careful probing around ‘challenges’. The Karen words used to describe the meaning of ‘challenges’ and/or ‘vulnerability’ included: barrier (ta-ti-ta), burden (ta-wee-ta-yoh), and a heavy load to carry (ta-wee-kher). In Thai ‘vulnerability’ directly translated as ‘fragile’ (phro-bang), and while it is formally used when describing a vulnerable population, we used the more neutral term for ‘difficulties’ in Thai interviews. Throughout the data collection conducted in local languages, the word ‘Sayar or Sayarma’ in Burmese, and ‘Thara or Tharamu’ in Karen were used by participants when referring to any clinic staff, frontline healthcare workers, doctors, researchers, and supporting team members. These salutations mean ‘teacher’ and are commonly used as a show of respect. Every interview session was audio recorded with consent, then transcribed and translated into English. Any ambiguities in original languages were thoroughly discussed across the team to verify what participants meant to say, with considerations of the meanings in social and cultural context. The transcript of each IDI and FGD, after de-identification, were imported into NVivo for data management and coding.

In addition to primary qualitative data collection, we conducted a participatory visual methods (PVM) workshop in June 2019, with 9 members of the Tak Province Community Ethics Advisory Board (T-CAB) [20], and 5 frontline healthcare workers (FHWs), to gain deeper insights and to test/confirm the themes emerging from our qualitative data. The use of drawings and stories offered additional ways for participants to creatively share their experiences and views on the role of research in their community [20, 28, 29]. Discussions, drawings, and co-scripted video messages were used to engage participants to share stories on the key research ethics themes of REACH and informed a final video, co-produced with participants. The video can be viewed here: REACH-In Our Voices [30]. Several drawings are included in results to further illustrate key themes.

### Data analysis

We conducted thematic, discourse analysis, focusing on ethical/moral aspects of the conversations with attention to the sociocultural context of the research encounter and daily life. We began with iterative open coding and team discussions, using NVivo software to organize, manage, and code the data [31]. We created an initial coding guide, based on a team review of selected transcripts, and iteratively tested and revised the coding guide with subsequent transcripts. Two Thai-Karen native-speaker team members, with professional proficiency in Burmese language, double-coded all transcripts with periodic discussions amongst the wider team when new codes were suggested or revised. A secondary ethnographic analysis of codes/content related to *Arr-nar/Kreng-jai* was conducted for purposes of this paper; the coding was done collectively by NK, SA, SN and NJ with support from other members of the team (JR, PYC, MK). This involved further discussion, reading, translation and analysis of these concepts and their meaning in ethnographic context. To benefit from multiple data sources and perspectives, our analysis also draws on our experience of the PVM workshop and visual outputs as described in study design and data collection [29].

### Ethnographic background: *Arr-nar/Kreng-jai* in the Thai-Myanmar border region

To help characterize the rich cultural norms of social behaviour relating to research participation in this region, we rely on the only available ethnographic analysis of two closely related concepts from Burmese, Karen, and Thai cultural traditions: *Arr-nar* in Burmese and Karen, and *Kreng-jai* in Thai [15].

In Thai, the direct translation of *Kreng-jai* would be ‘fear-heart’. The word ‘*Kreng*’ denotes fear, or a feeling of awe, and ‘*Jai*’ means heart. An ethnographic discourse analysis conducted by Wyatt and Promkandorn revealed that *Kreng-jai* could have as many as 26 different meanings [15]. The concepts encompass moral and social norms of behaviour, aspirational ways of being in the world, and one’s character or state of mind. An anthropologist and professor in law and anthropology, William J. Klausner had regarded the Thai cultural *Kreng-jai* concept as one of the most difficult for foreigners to comprehend [32].

The closely related concept in Burmese, *Arr-nar*, cannot be separated into two words and translated directly. *Arr-nar* is used widely and understood in all Karen dialects although the term is written in Burmese. While there is no direct Karen translation, the concept and the meanings are similar for both Karen and Burmese languages. Like *Kreng-jai*, *Arr-nar* cannot be reduced to a single idea. The nearest English translations encompass multiple meanings, depending on the social context, such as, consideration for others, being reserved and polite, feelings of gratitude and reciprocity, not wanting to burden or trouble others, graciousness and conflict avoidance, including polite refusal.

**Fig 1:**
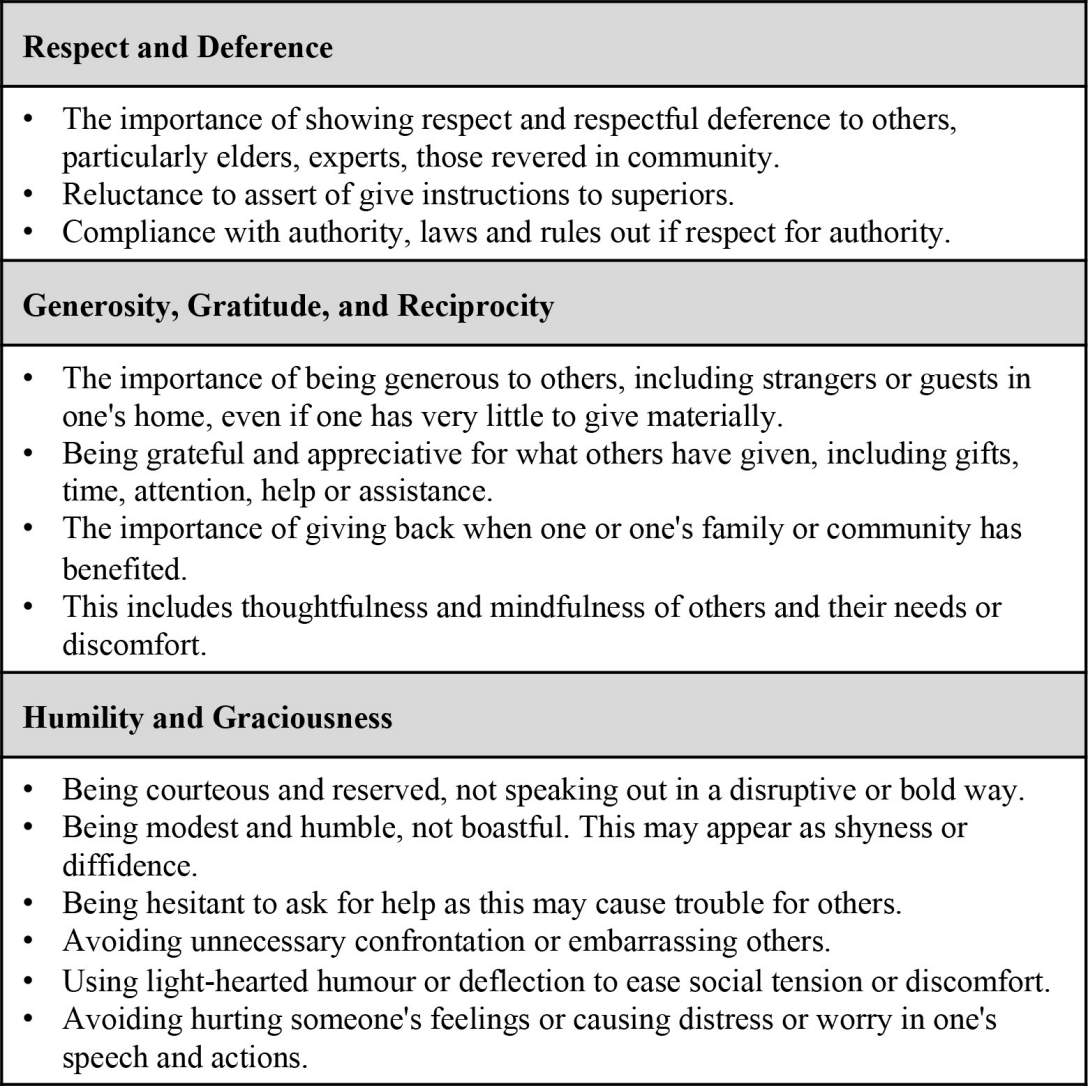
Multiple meanings of *Arr-Nar/Kreng-jai* in English terms.

In Fig 1, we offer a summary of the range of complex meanings translated from Thai, Burmese, and Karen to English, an adaptation from the discourse analysis conducted by Wyatt and Promkandorn with our own analysis and experience of the Thai, Karen, and Burmese meanings. These are approximate meanings that helped guide our qualitative analysis and discussion of the data in research ethics, including the relevant social contexts and relationships which can alter meanings and one’s ethical obligations. *Arr-nar/Kreng-jai* is best understood as a way of life, encompassing a range of norms of behaviour and orientation to others. These norms are taught to all children (both boys and girls) and expected to be practised by everyone throughout their lifetime. The concepts are not inherently gendered; the social obligations are meant to apply equally to men and women. As with any social norms, however, *Arr-nar/Kreng-jai* can be expressed differently by each individual, depending on their upbringing and life experiences and may intersect in subtle ways with other gendered roles in society. Similarly, as relational, social norms, they may intersect with power dynamics between people and groups. In presenting our results, we explore these norms within the research and health services context of our study.

### Practical research ethics framework

**Fig 2:**
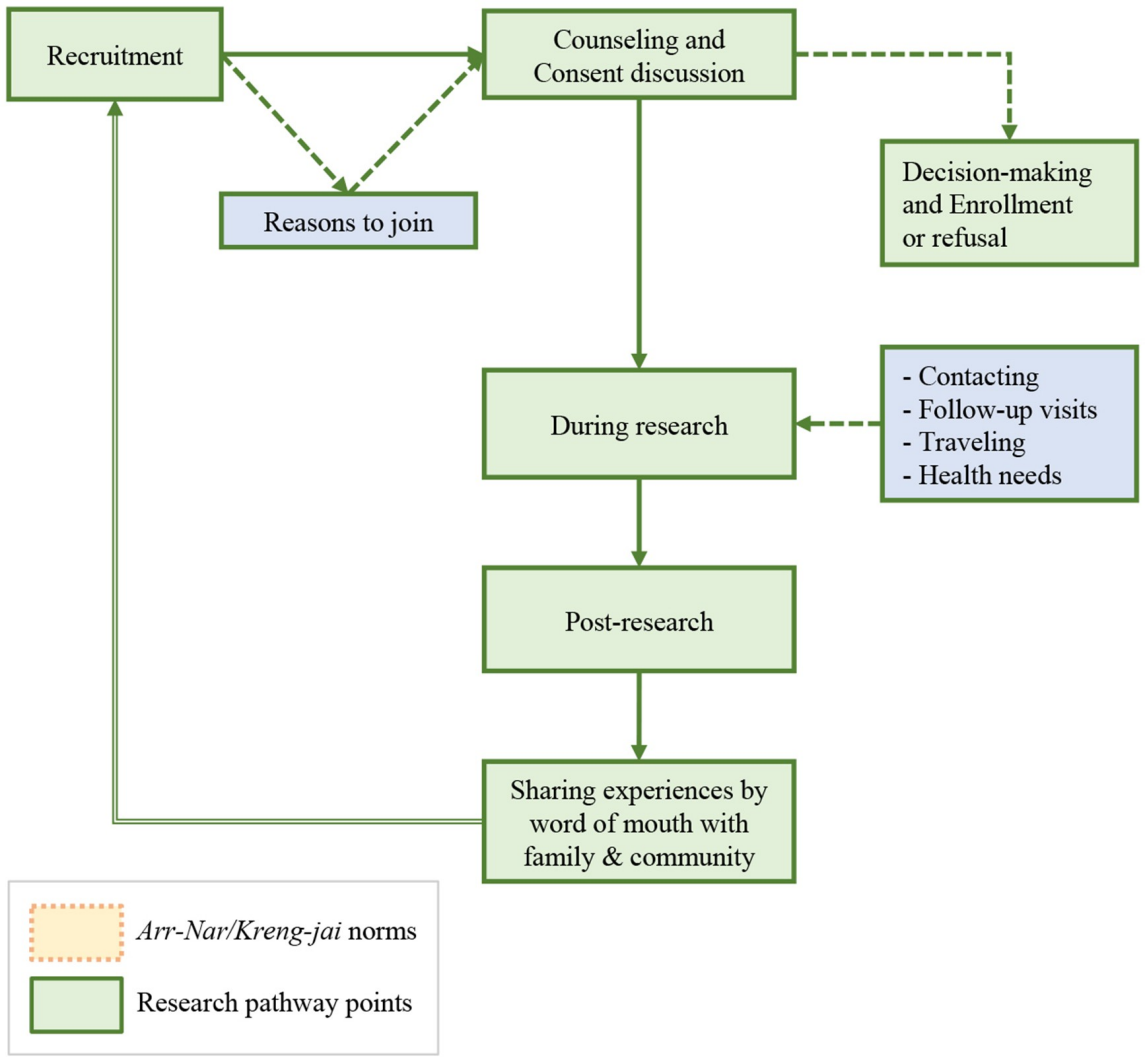
Key research encounters along the research pathway.

Practical ethics has historically sought to understand the complex moral worlds and dilemmas facing real people, viewing moral norms, principles and beliefs reflexively as emerging from the interaction of ethical theory and human experience [33, 34]. To situate ethical considerations in research within the complex cultural border context and with an eye to practical relevance for day-to-day research practices, we organized our analysis around key encounters between participants and researchers throughout the combined care-seeking and research pathways, common in many clinical research programs. The pathway maps our linked research studies with pregnant women at SMRU (Fig 2), where study recruitment occurred alongside provision of antenatal services. In the discussion section, we visually summarize results across this pathway, illustrating in detail, how socio-ethical norms of *Arr-nar/Kreng-jai* inform ethical responsibilities, such as ensuring understanding, voluntariness, or duty of care, and moral feelings, such as trust, appreciation, guilt or concern.

## Results

We begin with our observations of how complex sociocultural meanings of *Arr-nar* or *Kreng-jai* arise in daily living and research/health services encounters in this border context. We then explore, through our results and using the research pathway model (Fig 2), how these norms manifested along the research pathway from recruitment and consent through the various activities of study participation. We provide illustrative quotes, noting anonymized participant reference numbers and groups, indicating those from individual vs. group discussions.

### Understanding *Arr-nar/Kreng-jai* in the health services and research context

**Fig 3:**
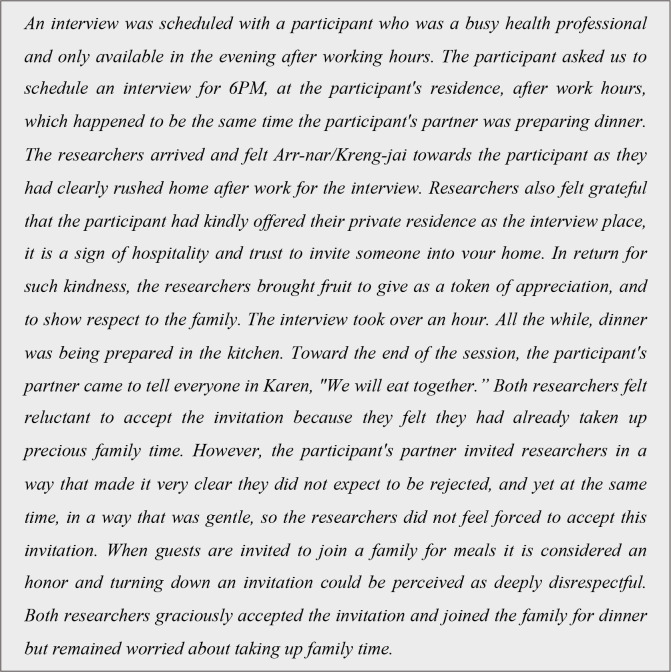
Illustrating how Arr-nar/Kreng-jai manifests in typical research encounters.

At its heart *Arr-nar/Kreng-jai* is a relational set of norms about how we ought to treat others and how we ought to behave in social encounters. It is often understood as a social orientation toward others or toward a ‘right path’. A person can be more or less socially oriented in the right way and direction toward *Arr-nar/Kreng-jai* or oriented away from, or against it. In the research context we discovered that these norms operate across social encounters between researchers and clinical staff, and between community members, participants, and their family members. In the context of a research study relations of social position, knowledge, and professional or community roles generate certain feelings and obligations toward others in the encounter. Participants and frontline researchers–mostly local Karen residents–spoke about ethical obligations in relational and often emotional terms. The values embedded within the complex concept of *Arr-nar/Kreng-jai*, as it emerged from our data, can be further analysed and clustered according to generally positive norms as well as concerns or worries about what happens if one does not properly orient toward *Arr-nar/Kreng-jai*. An illustration from our own research team’s experience is offered in Fig 3 below.

### *Arr-nar/Kreng-jai* and reasons for participating in research

Understanding why someone participates in research is ethically significant. Knowing someone’s motivation for joining a study helps evaluate whether the study offers important and fair benefits, whether the benefits of joining outweigh inconveniences or risks, whether the research is potentially coercive and whether studies are viewed as offering wider social value for the community. When participants were asked about why they joined the linked studies they often appealed to *Arr-nar/Kreng-jai* as an explanation, and when pressed, shared several interrelated reasons. These included believing they would receive important care and services for themselves and their baby, helping others, and giving back to the institution which they believe has brought benefit to the community. After joining the study, many remained in the study out of a sense of responsibility.

### Joining research to seek healthcare and information

One key informant, who has lived and worked in the area for more than 30 years, said there is one question women often ask themselves, “where am I going to deliver my baby?”, reflecting the limited choices for migrants in this region. Over many years, as trust has been built with local communities, women living in the border region have chosen to come to SMRU’s clinics to deliver their children. Women described traveling long distances on often muddy flooded roads, and risking border checkpoints when crossing into Thailand from Myanmar. They believed that they would be well looked after, and attending appointments would allow them more time with healthcare staff or doctors, benefitting themselves, their pregnancy, and their baby.

*Since I became pregnant*, *until I gave birth to my child*, *these are all supports [for me]*. *Whenever I come [here] they always help me*. *They support the health of my son; they look after him and see if his brain is good or not*. *They also support me in everything*. (P13, linked-study participant)

Through word-of-mouth from family members, neighbours, friends, and for some from their employers, SMRU became known among this border community for being a fever clinic and clinic for pregnant women that they can rely on for care. Most migrants in this area are undocumented ‘cross-border’ and ‘unstable’ migrants, and as such, they are unable to afford and eligible to healthcare services in Thai government facilities.

*For me…why do people come to the clinic? It is because it is also for my health*, *and also for my children*, *that’s why*. *…I just have to come here*. *It is like the place I can rely on*. (P15, linked-study participant)

*My son had heart disease and got referred to [name] hospital*. *We don’t have money to pay for his treatment and without joining research*, *how could we arrive there*? (P34, partner of linked-study participant)

**Fig 4:**
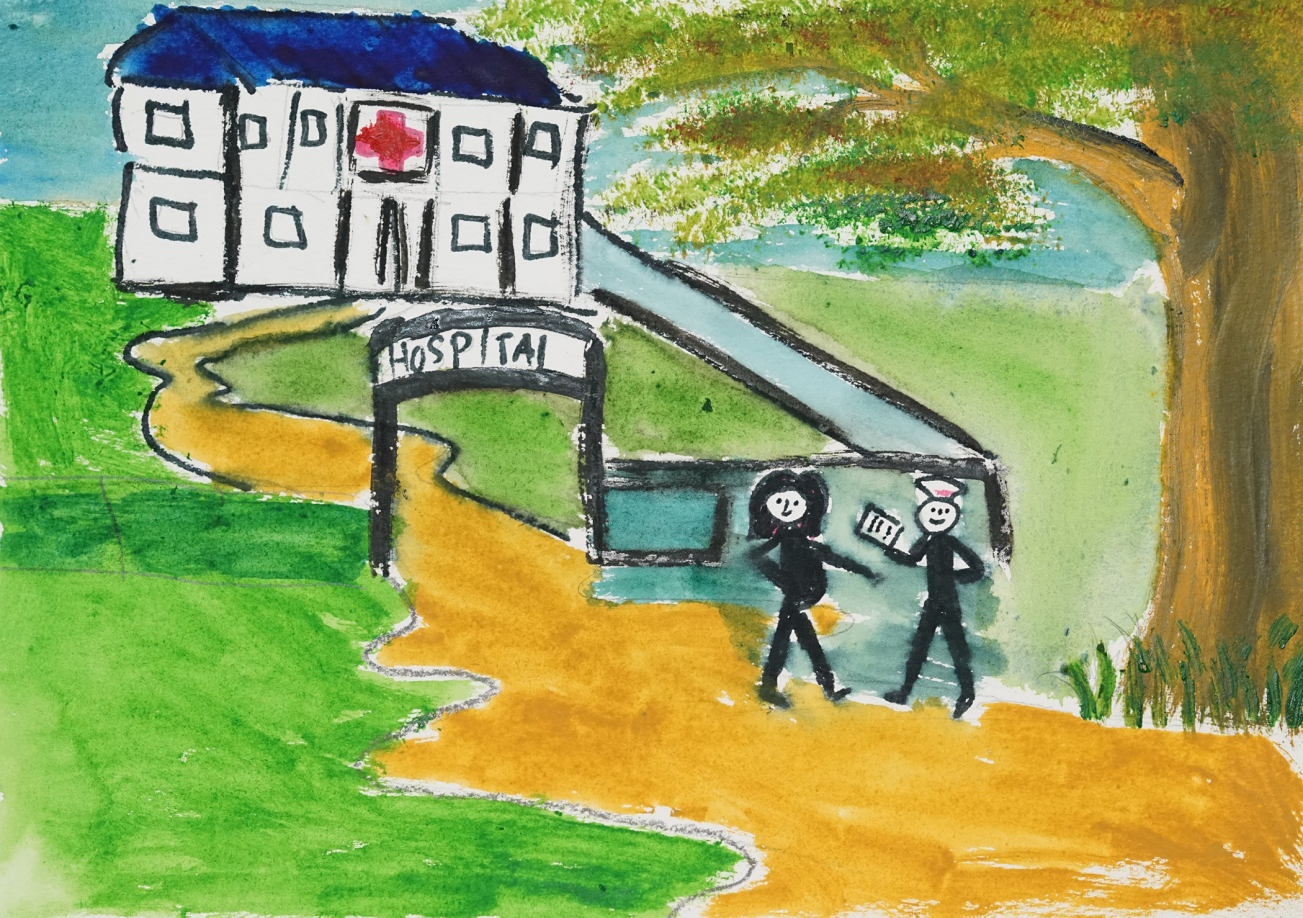
Illustration of pregnant woman speaking with staff about a study, hand-drawn by REACH PVM participants.

Research information, the potential benefits of participation, and health information were often seen by women as interlinked, given that research pathways closely aligned with health services and the provision of care. This theme was illustrated by a drawing by PVM participants, showing a researcher meeting a pregnant woman with useful health information (Fig 4).

### Joining research to help others

Women described participation in research not only as a way to receive health services and information, but also to “help others” from their own community through wider health improvements or by contributing to health knowledge that might benefit others, their communities, and beyond.

*Interviewer*: *For you to decide to join the study*, *was it easy or difficult? Did you think about it for long*?

*P13*: *I think it was not difficult…*. *I joined*.*… What they wanted to know they [researcher/doctor] will get to know…. They will know about this disease and they can look at it and how we got it and they can help others also*.…

*Interviewer*: *What if you don’t get any benefit*?

*P13*: *Doesn’t matter*. (P13, linked-study participant)

Participants shared similar reasons for deciding to take part in our REACH study, a social science and research ethics study, quite different from the linked clinical studies and offering no direct health benefit for them. Some viewed our study as an opportunity to share stories about their daily lives and experiences surrounding care-seeking and research participation. They expressed that by participating, others could learn from their experiences.

*As a human*, *we have gone though many things in our lives*. *It is about our feelings*, *challenges*, *problems*, *poverty*. *What we share*, *it will also help other people to learn about us*. (P15, linked-study participant)

### Joining research out of a sense of gratitude and trust

As SMRU has been providing humanitarian aid, clinical services, and conducting research for decades in the region, many participants expressed feeling of “*Arr-nar/Kreng-jai*” towards the institution, its doctors, and staff, based on the wider reputation of the institution in the community or prior personal experience as a reliable place for assistance, care, and information.

Previous personal experience was an important factor. Many women interviewed had previously delivered their children at SMRU prior to joining the clinical study, while a few were visiting SMRU for the first time, but had heard about the facility through other mothers.

*For myself*, *at first*, *I did not dare to come*. *I asked other people where they go for antenatal care*.*… I asked my sister to accompany me to show me the place [SMRU’s clinic]*. *That’s how I went*.*… I delivered my child [and] he was big*, *around 3 kg*. *They take good care of me regularly*. (P4, linked-study participant)

*I feel good*, *that is why I come here*. *The staff here are nice; good communication*. *Sometimes*, *I feel like I was healed because they speak to me nicely*. (P16, linked-study participant)

**Fig 5:**
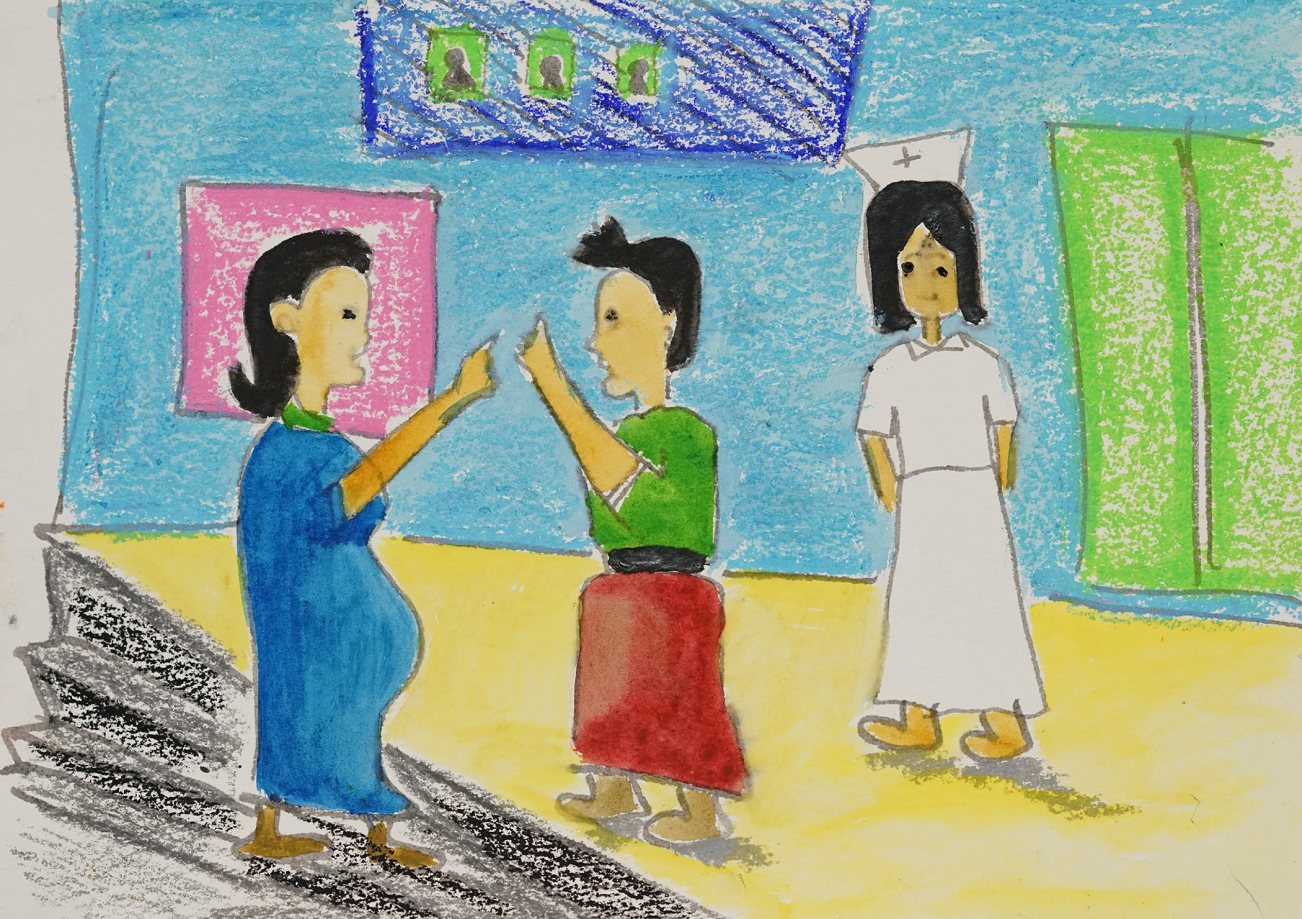
Illustration of women sharing information with other women, hand-drawn by REACH PVM participants.

Some pregnant women described bonding with each other through the research experience. They came to know each other through participating in one of the SMRU clinical studies. Naturally, they became more acquainted as the study advanced, and often travelled together for follow-up visits, encouraging each other to stay in the clinical study even when journeys were difficult. They encouraged other pregnant women from their village or community to attend early antenatal care. As migrant women, they faced similar challenges in accessing health information and care, and by sharing information women expressed hope that other pregnant women, mothers, and children will be able to access quality care, as illustrated in a drawing by our PVM participants (Fig 5).

### Remaining in research out of a sense of responsibility

Reasons for remaining in an ongoing study can be as revealing as reasons to join a study. Research participants in this border region often go to great lengths to return for study follow-up appointments and expressed strong feelings of responsibility to turn up for their appointment.

**Fig 6:**
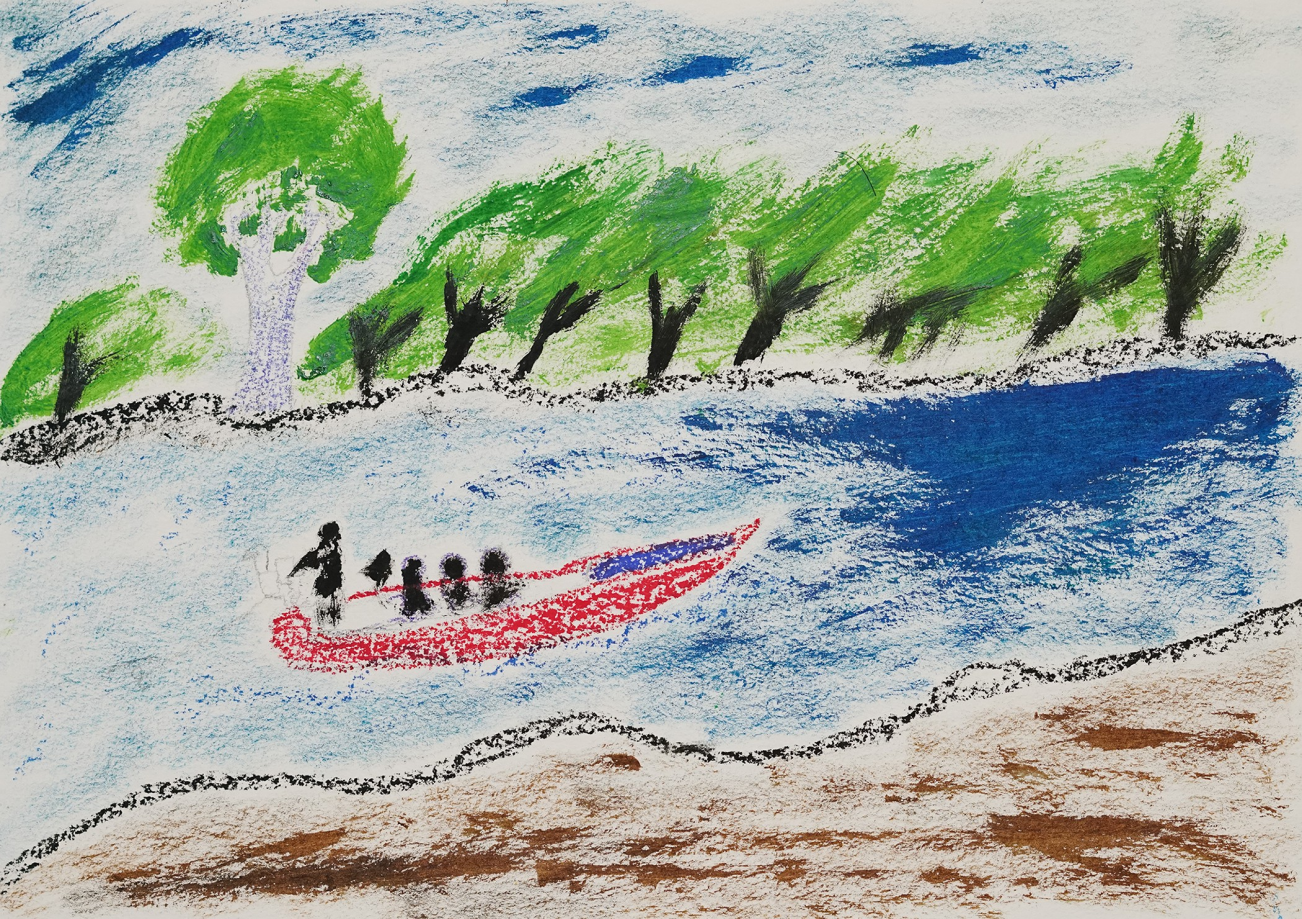
Illustration of travelling risks, hand-drawn by REACH PVM participants.

The women reported that out of respect they disregard traveling risks (e.g., at border check points) and leave the safety and obligations of home or work, trying their best to return for their clinical or study appointments. Several participants illustrated the challenging, often epic, journeys to the study site during rainy season, as we see in this drawing by our PVM participants (Fig 6).

Using the local respectful address, ‘Tharamu’, participants explained that remaining in a study and turning up for all appointments, despite hardships, shows respect for researchers waiting for them at each appointment.

*P8*: *For me*, *I always come back*. *I delivered my baby here*. *Tharamu called me once a month*, *once a week*, *I always came*, *I was never absent from study*. *I always brought my daughter here*.

*P7*: *When Tharamu ask us to come*, *when we have work*. *We just leave our work and come*.

*P8*: *We leave*, *and really come*.

*P7*: *I don’t think of anything else*…

*P8*: *Yes*, *just leave [work] and come*. *We have to respect each other*, *right*? (FGD, linked-study participants)

Importantly, as expressed in P8’s last comment, obligations of respectful behaviour was viewed as mutual respect, not merely as a respect owed to authority. The researchers we interviewed also remarked on the strong commitment observed among participants, as described here:

*What makes a woman get on the back of the motorbike when she doesn’t feel well to come all the way here? And she said*, *she just wanted to help*. *It’s incredible… I thought*, *what drives these people to come*?*… They risk their lives because they had to cross the river…and she said she just wanted to come*. *She thought that it was her duty to come*. *It was a commitment she made*. *The other woman said the same thing*. (FGD, frontline researchers)

### Implications of *Arr-nar/Kreng-jai* for assessing voluntariness, understanding and refusals

A core principle of ethical research requires that participation be voluntary. In the most basic sense this requires that a participant not be unduly influenced or coerced by someone else to join a study. When assessing voluntariness in research, if voluntariness is thought narrowly of as an individual person’s will, it can be challenging to account for strong social influences on a decision to participate, especially in more communal societies governed by sociocultural moral norms like *Arr-nar/Kreng-jai*.

### Social influence and *Arr-nar/Kreng-jai*

As illustrated in the earlier scenario (Fig 3) where researchers felt if it would be impolite to decline a dinner invitation from a research participant, *Arr-nar/Kreng-jai* informs all daily living practices, wherein one should not inconsiderately decline invitations. An invitation to participate in research, coming from someone who is seen as important, busy, or offering other valuable services to the community, can elicit the same response. For SMRU’s research context, *Arr-nar/Kreng-jai* was strongly observed amongst participants, who expressed feelings of gratitude, appreciation and respect toward the institution, researchers, and frontline healthcare workers for healthcare services received. This raised an important ethical question: had participants joined research studies purely based on their interest in participating, or did they agree because they felt *Arr-nar/Kreng-jai*, either not wanting to be impolite by refusing to participate, or feeling a deep sense of social obligation to participate? This question raised concerns around understanding and, potentially, voluntariness.

### Concerns about assessing understanding

There was some evidence that politeness and social deference might be a barrier to ensuring participant understanding of study information. Some research participants admitted they would not ask questions if something was unclear because they worried about burdening staff and wanted to be mindful toward the staff who are performing their duties—asking questions was seen as creating more work for staff.

*P4*: *I wouldn’t dare to ask*.

*Interviewer*: *How about me right now…would you dare to ask me*?

*P4*: *Yes*, *when I’m alone*, *without other participants… I dare to ask*.

*Interviewer*: *What was the reason when you do not dare to ask*?

*P4*: *You know*, *it’s like feeling ‘Arr-nar*’ (P4, linked-study participant)

Most studies conducted at SMRU are biomedical studies on malaria, maternal and child health, hepatitis B, prevention of mother-to-child transmission of infectious disease, scrub typhus, and melioidosis. The scientific parts of these studies can be challenging to translate and explain in local languages. Information sheets and consent forms are long once translated, and literacy rates are exceptionally low. A reluctance to ask questions due to *Arr-nar/Kreng-jai* magnifies challenges for the study team to ensure that participants adequately understand the study.

### What does refusal look like within a culture of *Arr-nar/Kreng-jai*?

When asked about the issue of voluntary participation, participants clarified that they did not feel like they were compelled by researchers or family members (e.g., husbands) to say yes. They understood that they could decide *not* to join the study, and that there would be no impact on their ability to receive care at SMRU’s facilities. Additionally, they felt the information provided during the consent process would allow them to foresee and anticipate health benefits and possible burdens from taking part in research.

*It has benefit*, *for myself*: *I will not have that disease anymore*. *For me*, *it’s like*, *I have an interest for things that are being studied*. *For me to join*, *for me to be invited to join*, *I am not reluctant to join*, *I am happy to join*. (P58, linked-study participant)

Some women explained how they can say ‘no’ to being in a study through more indirect or polite refusals, consistent with *Arr-nar/Kreng-jai*. Often, the reasons given for not wanting to join included bad roads, severe weather, needs of family members, work obligations, or being too busy with other important tasks. In some cases, they agreed to join research out of politeness with no intention to participate and would later offer an excuse. They refused, without refusing.

### How *Arr-nar/Kreng-jai* can make it difficult to assess research burdens and benefits

Another central ethical requirement for responsible research, especially with vulnerable or marginalized populations, is that the research offer fair benefits and compensation for burdens (e.g., travel), and longer-term benefits to the community, such as health improvements and disease prevention. It was clear from women participating in research related to pregnancy that they valued the benefits of clinical service and peace of mind in having a safe place to deliver their babies. While the women we spoke with did not report experiencing any direct risks when taking part in the research they were enrolled in at the time, they did share that participation meant extra trips for follow-up visits, explaining that these journeys can be long and sometimes risky as migrants. However, most participants described long journeys and risks related to border-crossings matter-of-factly; only a few participants admitted that this was burdensome. For this reason, they did not see reason to raise concerns about travel. Instead, it was the frontline healthcare workers, many from the Karen community, who appreciated and spoke about the hidden hardships involved in research participation:

*P32*: *Not only do they lose a day of wages*, *but they have to find the money to pay to get there*. *So*, *they might lose that day*, *plus two weeks of wages to get there…*. *It depends on how far away they live*. *It’s a huge financial loss*.

*P47*: *With migrant workers*, *when they come to the clinic*, *they have to pass the checkpoint*, *right? So*, *also there’s the risk of being caught by the police*. (FGD, frontline researchers)

Even when asked directly, participants were not forthcoming about extra difficulties caused by travel, border checks, or leaving the fields or family duties, so researchers and frontline healthcare workers sometimes found it difficult to gauge when benefits were genuinely adequate and fair, to truly mitigate or compensate for hidden risks or burdens. Researchers we spoke with were confident in the longer-term benefits of their research for directly helping migrant women, but worried about whether wider benefits, such as improved interventions for malaria in pregnancy, were doing enough to address participant needs in the immediate term, even feeling guilty about professional gains from research, as these two researchers shared:

*P70*: *The idea of the TDF study*, *if it works*, *it will have a very big impact on pregnant women with hepatitis B*. *Could really prevent mother-to-child transmission*, *in a better way than what we’re capable of at this moment*.

*P32*: *But we also use the patients*, *I use the patients*, *you know [very soft voice]*, *I use them*. *They get 500 baht [$14*.*70]*, *I get their blood*, *I get a PhD*, *I get my papers*. *What do they get from what I have done to them? They don’t get much [very soft voice]*. *And*, *for the urine check*, *they get a 100 baht [$3*.*00]*, *80 baht [$2*.*21] …and that makes me feel pretty guilty*, *I think*. (FGD, frontline researchers)

Norms of reciprocity and fairness, deeply rooted in *Arr-nar/Kreng-jai*, were particularly strong amongst frontline health workers, who were also from the migrant Karen border villages, as they sympathized with the complex health and social needs of either patients or research participants.

*We also sympathize for them*, *when they say…we don’t know what to do*, *[but] we are helping each other*. *That’s what I say*, *I am here*, *they always come here*, *and I always look after them*. *We have to help them*, *they also help us; that’s what we tell them*. (P36, frontline healthcare worker)

Feelings of concern or guilt for not doing enough are core to regional norms of *Arr-nar/Kreng-jai* and were expressed by both Karen ethnic frontline staff and international staff who had been living and working here for a decade or more. This sentiment extended to patients who do not qualify for research and are not eligible for study benefits. Frontline staff shared that they try to provide equal care for all without privileging research participants, but sometimes, compensation that is being offered to research participants for their trouble and not to regular patients results in unintended disparities and causes distress for staff. To them, this was viewed as a violation of *Arr-nar/Kreng-jai*.

*If we look at it*, *all of them were sick but one person was sick with other disease*, *another person was sick with malaria*, *and another person could have pneumonia*. *They were all in serious condition but the person who joined [the study] and had malaria would be given a gift while the other person would not receive anything*.*… I think for some*, *they could not afford to buy even soft drink and there was no gift for them*. (P26, frontline healthcare worker)

Concern about offering enough for patients and participants alike was sometimes exacerbated when there were limited resources for providing humanitarian services for everyone living on the margins. In this way, the obligations assumed when orienting oneself to *Arr-nar/Kreng-jai* can create a sense of moral distress for frontline staff—believing it was right to do more but feeling unable or constrained to respond adequately to widespread needs for all patients. Some staff described the emotional challenge of being in dual roles as clinicians and researchers when, in their researcher role, community-wide benefits from studies were not immediate; in the short-term there were so many health and social needs that could not be met through research. Researchers’ feelings of wanting to give more was heightened in the face of so many participants who dutifully came for research follow-up visits out of a sense of responsibility and mutual respect. They worried that *Arr-nar/Kreng-jai* could, at times, mask greater need for compensation, when some participants joined despite personal costs, out of a sense of duty or responsibility.

*P40*: *For some it cost 500 baht [****$****14] for a round trip and the study compensates 300 baht [****$****9]*. *The other 200 baht [****$****5] is out of pocket*. *When that happens sometimes*, *they don’t want to come for follow up anymore*, *but some feel Arr-nar for staff and still come*…

*P37*: *For them to come back for follow up*, *there’s also the expenses that will cost them; that’s another thing*. *That’s why*, *for us to do the work*, *we have to understand all aspects [affecting] them*. *We also have to look and understand their situation*. (FGD, frontline healthcare workers)

## Discussion

Research ethics guidance for international sites like the Thai-Myanmar border region comes from multiple sources, including international and country-level guidelines and principles and local oversight from local ethics committees. Guidelines include high-level, often abstract, ethical principles, many still reflecting European or North American ethical traditions that must be interpreted by ethics committees and researchers in practice within diverse cultural contexts. All international researchers and many local researchers, will have received research ethics training informed by these guidelines, and yet learn through experience the additional complex norms and relational obligations at play in everyday research practice.

**Fig 7:**
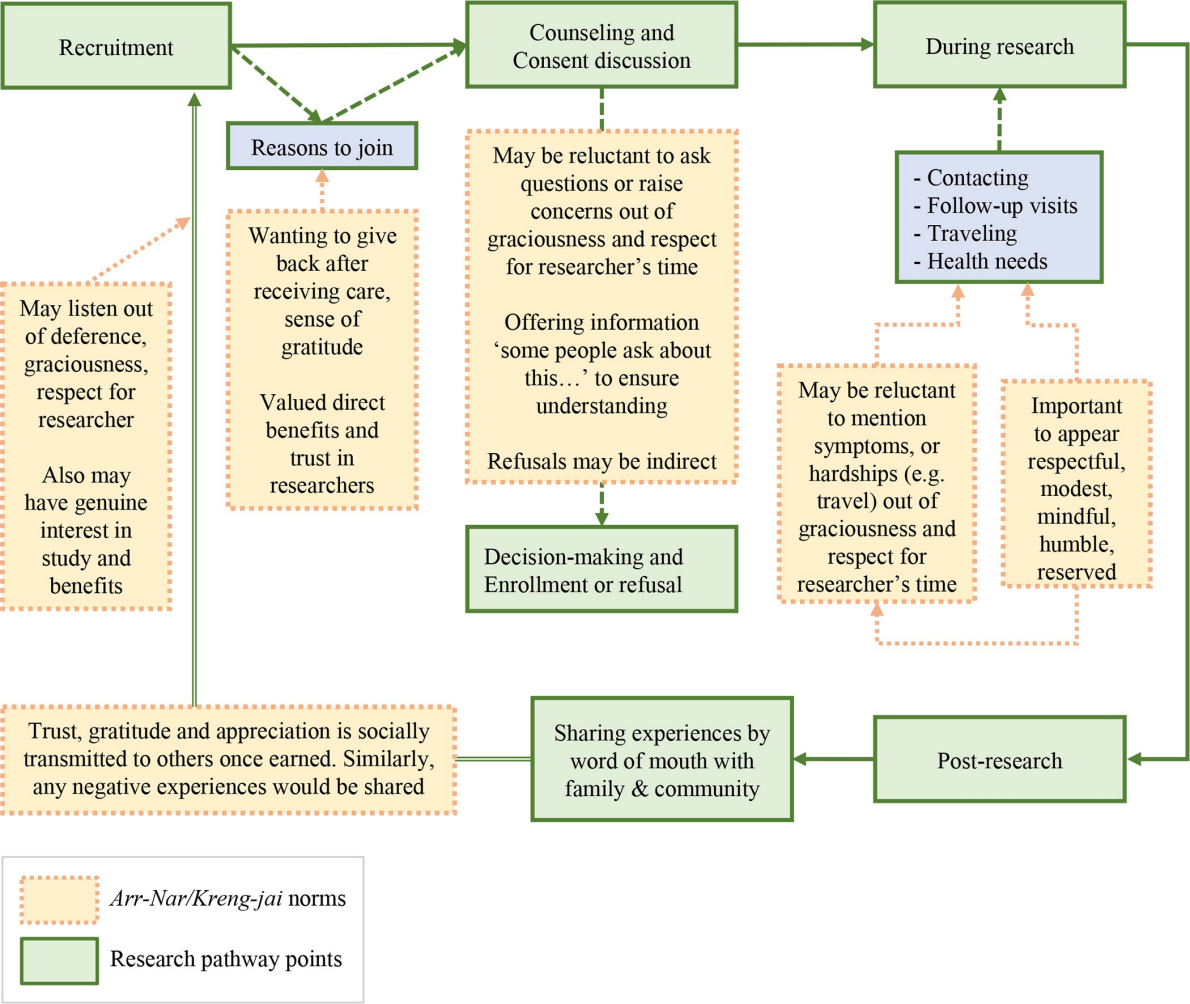
Ethical dimensions of *Arr-nar/Kreng-jai* in researcher-participant encounters.

We have studied one such sociocultural context to illustrate how greater sensitivity to subtler moral and social norms can be central to ensuring the ethical conduct of international research. We have shown how norms of *Arr-nar/Kreng-Jai* overlay key encounters along the integrated healthcare and research pathway and inform the behaviour and responsibilities of both participants and researchers, as summarized below in Fig 7. These conversations helped reveal hidden aspects of participants’ struggles and expectations, as well as points where potential misunderstandings might be avoided. This can serve as a practical guide, illustrating how the ethical and social norms of *Arr-nar/Kreng-jai* inform ethical commitments, feelings, and communication between the participants/community and the researchers/research institution.

A significant ethical issue that emerged in this context is that norms of *Arr-nar/Kreng-jai* can manifest in hidden burdens, where a person might not complain to avoid being troublesome or causing disturbance by asking for help. For example, when a participant feels *Arr-nar/Kreng-jai* to ask for clarification of anything they do not fully understand, the feeling of *Arr-nar/Kreng-jai* outweighs their need to have their question addressed. Similarly, a participant who experiences hardships during travel for research might be deeply reluctant to share these difficulties out of *Arr-nar/Kreng-jai*, or deference and respect for busy researchers and health care staff. Within norms of graciousness and respect for clinical and research staff participants may have questions, worries, or be experiencing hardships—like costs of travel—that are not shared without gentle prompting, “reading between the lines” of what is being said, or observing body language. This has also been documented in the Kenyan context by Njue and colleagues who noted that as researchers we often underestimate costs to participants, particularly worrisome in under-resourced settings where even small costs can be monumental for struggling families [11]. Knowing this may help prime staff to be gently persistent, as they are also held to the same sociocultural norms of politeness and non-conflict. It can be culturally appropriate to probe with genuine concern about wanting to help someone or understand their situation. Sharing information that is commonly needed without prompting, and not taking a lack of questions as not wanting to know more, can help ensure a meaningful consent process in this and similar contexts. In terms of practical research ethics, listening and observing are as important, if not more important, than talking.

The institutional arrangements within our case study offer an important example of situating a long-standing research, humanitarian, and clinical services programme in a politically dynamic border region, where migrants with limited access to essential health services through either the Myanmar or Thai governments especially need and value free clinical care [35]. There are strong ethical reasons for setting up international health programmes to provide clinical services and humanitarian aid, with research instrumentally aimed at serving the needs of the local population [36]. Findings in our study illustrate how important ‘earned trust’ is throughout the community, and how trust is shared across women’s networks and community networks, through word-of-mouth. At the same time, it is important to constantly evaluate the meaningfulness of voluntary participation and fair benefits in research occurring in contexts of poverty and political vulnerability, particularly against such strong norms of gratitude, duty, and graciousness.

Specifically, it is important to consider how research programmes working in contexts of structural vulnerabilities and deeply held norms of graciousness and gratitude can strike the right balance between offering fair benefits to participants and the community and respond to the possible risks and burdens of research participation, when local moral and social norms may make such ethical assessments difficult. In a cultural context where strong norms of gratitude and reciprocity guide people’s decisions to join research, and where access to public health services is limited, one might question whether agreement to participate in research represents what some have characterized as ‘an empty choice’ or undue influence—not meaningfully being able to refuse participation given economic hardship [37, 38]. While this is an important concern, in our setting, acting from moral reasons to ‘give back’, acting out of reasons of trust after having received valuable care, and after undergoing robust engagement and consent, could be interpreted as exercising agency and real choice based on core communal values. The social aspect is critical to appreciating how ethical reciprocity works in this setting. Trusted social networks are a key feature of our context and if an institution were not trusted, they would likely have difficulty engaging patients or recruiting research participants, encountering a formidable wall of polite but firm avoidance.

Despite serious political and economic vulnerability amongst the Karen migrant community, we observed that there is also power in their social networks, where valuable information is shared and trust (or mistrust and concerns) are readily transmitted throughout the communities. The women in our groups also described relationships formed over time between migrants and clinical and research staff, not as relationships based on humanitarianism as “rescue”, but rather a more empowering and bidirectional relationship where members of the community see themselves as also giving back, giving their time and health information to help others in their community. This moral self-perception, deeply rooted in an orientation toward *Arr-nar/Kreng-jai*, challenges common perceptions by outsiders of Karen migrant women as a wholly vulnerable population and overlooks an important aspect of their moral agency. The sense amongst these women was very much that if a researcher or research institution is not worthy of their trust, time, and energy, they would not participate or withdraw. This observation echoes work by Roest, Nkosi and colleagues on the subtleties of respecting a person’s agency in research participation within the constraints of often severe structural vulnerabilities [39].

Not compensating sufficiently to mitigate hidden burdens of participation, however, remains a serious worry. Considering some of the cultural difficulties of determining whether individual needs are met through research, it may be that engaging community networks through discussion about issues such as fair benefits and hidden burdens of research, rather than pressing individuals to say something that is socially difficult, might be a more effective way of finding out what is really needed by the community. Community consultation has in fact been effective for assessing fair benefits in the coastal Kenyan context [11, 40], and for engaging community knowledge and action around broader public health surveillance and vaccination efforts in our own border setting [20].

On the individual level it might also be the case that voluntariness simply looks different and less overt. We observed clear examples of polite refusals in our study, such as giving excuses for not being able to join or return to a follow-up appointment. Such refusals show no less voluntariness than a participant who reads a consent form and says directly, “no thank you; I’m not interested in joining this study” [14]. This echoes work done by Kamuya and colleagues who describe how Kenyan norms of *utu* in Kiswahili—which include politeness and conflict avoidance—influence research participation, often leading to what they describe as “silent refusals” in research in coastal Kenya [41–43]. Similarly, Nkosi and colleagues, describe how research participants in Kwa Zulu-Natal can be motivated by a similarly deep and complex cultural norm *Ubuntu* from the Nguni languages of South Africa, to participate in research out of a sense of solidarity if research is seen to benefit everyone in the community, or even to help a local researcher as a fellow South African. Individual refusals in this setting are often indirect, as they described one participant escaping out the back door when research staff came knocking, to avoid having to say ‘no’ to the researcher’s face [44]. And yet marginalized communities find power to engage collectively around health and social priorities.

This calls to mind James Scott’s idea of ‘every day resistance’, as resistance to oppression—might this sense of resistance offer a way of understanding polite refusals to participate in research? At the time our study took place the war in Myanmar, and violence against Karen Burmese along the Myanmar-Thai border was escalating. Encounters with border agents or military personnel may be met with such resistance. However, within the context of our study, where SMRU has served as a trusted refuge for many migrants needing safe health care through relative peace and wartime, this idea of resisting against the oppressor seems misplaced when considering the participant-clinician-researcher relationship. While there is an important power difference that ought to be taken into account, none of the participants or community members in our study expressed feelings of being oppressed by clinicians or researchers [45]. *Arr-nar/Kreng-jai*, *utu* and *ubuntu* are experienced by everyone in the community, as a sense of power and action through solidarity, which we believe better captures the feelings shared by participants in this context. The lesson for international research ethics is to broaden its focus on social values and relationships within community, where ethical obligations are understood better and embodied as deeply social and communal.

Research encounters will reflect the wider socio-cultural norms of the communities in which it takes place. As a way of life and orientation to others, *Arr-nar/Kreng-jai* may intersect with other social norms, power relations and gender roles in subtle ways. For example, the strong respect shown to researchers in our study, was explained in terms of perceived benefits and earned trust over time, although some did speak in more reverent tones about not wanting to trouble busy clinicians or researchers. We should not overlook the fact that research imposes its own hierarchies and relations of power—for example, between educated frontline health workers and participants with low literacy. While participants in our study did not explicitly speak to the nuances of power or gender roles, it would be important in any study to consider whether power differences or norms of deference could be felt more strongly by some due to social status or gender.

To what lengths should researchers and institutions go to create the conditions for more culturally responsive research ethics? Returning to the issue of meaningful refusals, do researchers have an obligation to create the culturally appropriate conditions under which people can say no without feeling this is wrong, to provide a more graceful ‘out’ from research? If so, how might this be built into ethical research recruitment? As far as cultural difference impacts and informs core ethical principles of research—such as voluntariness—it seems critical to try to address what is “lost in translation”. Certainly, we could improve culturally sensitive research ethics training to prime researchers to notice these subtleties of language, body language and behaviour. Further efforts might require building cultural engagement into funding applications, to ensure adequate time and resources.

The practical research ethics pathway we have presented can serve as a model to adapt for other settings, offering new, non-local or foreign researchers a cultural road map tailored to their context and created by local community members and research staff. While our mapping was informed by a longitudinal qualitative research ethics study, research units and teams might with less effort draw on the experience of local community members and staff to inform team discussions and training. For example, Bull and colleagues have evaluated how one might conduct a rapid cultural assessment for approaches to consent [46].

It would be a mistake to assume, though, that these recommendations are directed only to “foreign” or expatriate researchers. Researchers interviewed for our study were all long-serving staff, well embedded and living their lives in the border region for many years, even several decades. While we did see more pronounced, direct appeals to *Arr-nar/Kreng-jai* among the predominantly Karen health workers and frontline research staff, we also saw examples of regional and expatriate researchers expressing deep empathy toward community members, norms of reciprocity, graciousness, and even feelings of guilt when research benefits were not addressing immediate needs. These suggest points of common moral feeling across cultures that could be further explored through community engagement and cross-learning.

True, even for long-serving staff there are well known anthropological limits to how deeply outsiders can appreciate a culture, without having been raised or lived a long time within it. There are words and expressions like *Arr-nar/Kreng-jai* or *Ubuntu* that have such deep and complex meanings, that no foreign words can capture in simple translation [47]. As we have illustrated, the very meaning of such concepts shifts across different social situations, hierarchies, and relationships. Expressions of moral judgments, obligations, and meaning will go beyond words and encompass very subtle facial expressions, tone of voice, body language and demeanour. Is that so very different from any moral community the world over? Making a sincere attempt to bridge understanding, as we witnessed across this diverse team and community, seems an ethical commitment worth working toward.

An important limitation of our analysis is that we did not set out to conduct an ethnographic study on *Arr-nar/Kreng-jai* specifically, but rather, an investigation of ethical obligations when conducting research in contexts of structural, political, and socioeconomic hardships in diverse cultural settings [14]. In our data, reflexive notes, and group reflections, *Arr-nar/Kreng-jai* appeared everywhere, as “the elephant in the room”, until we realized the need for a direct analysis through this rich lens. No doubt we could have achieved greater depth of understanding with additional longitudinal ethnographic observations and targeted follow-up questions, explicitly aimed at understanding these concepts as well as intersecting norms around power and gender in the research context, beyond the reflections offered here. That said, as Thai-Karen lead researchers, of parents born in Myanmar, we very fortunately were able to draw upon deep personal insights into the norms and practices discussed here.

## Conclusions

Our findings not only have inherent interest for reflecting on and enriching our understanding of moral concepts central to research ethics, but also challenge the international research ethics community to consider diverse ways of understanding our shared obligations in research with local communities. By mapping sociocultural ethical norms of *Arr-nar/Kreng-jai* to common encounters along the research pathway, we have offered practical ethical guidance for researchers working in South East Asian and similar ethno-cultural contexts. We hope this model and our reflections inspire others to continue developing research ethics support that is more responsive to local and regional ethical norms.

## Supporting information

S1 Appendix Topic guide for IDI and FGD for Group 1a and 1b. (PDF)

S2 Appendix Topic guide for IDI and FGD for Group 2 and 3. (PDF)

S1 File COREQ_checklist. (PDF)

S2 File Alternative language abstract. Abstract in Burmese language.
(PDF)
