# Supplementary material for: Culturally responsive research ethics: How the socio-ethical norms of Arr-nar/Kreng-jai inform research participation at the Thai-Myanmar border
Source: PLOS Glob Public Health. 2023 May 4;3(5):e0001875. doi: 10.1371/journal.pgph.0001875 (PMC10159138; doi:10.1371/journal.pgph.0001875)
Supplement: S1 Appendix — (PDF) [file pgph.0001875.s001.pdf]

Topic Guide for Interviews and Focus Group Discussion
Group 1a& 1b

Warm-up and daily life [for IDIs only]:

1. Can you tell me a little about yourself and your family? How many family members? Where are you from? Where were you born?
2. Where do you live? How long have you live here? How far is it from here? How do you come to this place?
3. Where did you go to school? What languages can you speak, read or write?
4. What are your current daily activities?
5. What do you do for a living? What are your sources of livelihood? Do you have to send money home (if migrants)?

Warm-up [for FGDs only]:

1. We are speaking to you because your wife/ relative has been involved in a study around pregnancy – how old is the baby now/ when is the baby due?
2. What made you decide to take part in the discussion today?

Challenges, problems, difficulties in daily life

6. How are things going in your life in general? How would you describe your own and your family's health? Do you eat and sleep well? Are there any concerns about your or your family members' health?
7. Have you faced any other challenges/difficulties/hardship have you faced in your life (economic, political, social and cultural)? Can you tell me more about that?
8. Are there any problems in your community that impact on you/ your family negatively or that you worry about?

Sources of support, mitigating factors, coping, resilience:

9. When you face some challenges/difficulties/hardship, what do you do? How do you cope with them?
10. Have you sought support from anyone such as your family members, friends, relatives, and people in your community etc.?

APPROVED
ETHICS COMMITTEE
FACULTY OF TROPICAL MEDICINE
MAHIDOL UNIVERSITY

Digitally signed by Faculty of Tropical Medicine, Mahidol University
DN: c=TH, st=Bangkok, l=Ratchathewi, o=Faculty of Tropical Medicine, Mahidol University, cn=Faculty of Tropical Medicine, Mahidol University, email=tmectropmed@mahidol.ac.th
Date: 2018.11.01 16:04:35 +07'00'

11. What are some of the main sources of support in your life for example for your health, for work, family matters, child's education etc.?
12. What sources of support are most important to you and why?
13. When needed, where do you usually seek health care from? Why do you chose this/these place(s)?
14. Do you find non-medical support from this clinic/hospital and researchers here? If so, how have they helped you?

Research ethics – how is research adding benefit, addressing vulnerability, or adding to burdens:

15. How did taking part in research (DMA/ TDF) make you feel? How did you first hear about the study?
16. Why did you decide to participate? Did you make decision alone or was anyone involved in the decision (ask who to recruit husband or family members)?
17. Was it difficult to make the decision to participate? Why or why not?
18. How does participating in research affect your life? Are there any benefits or any negative consequences? (probe for extra supports, such as access to medical care)
19. Do you remember your experience at the beginning with the consent process? Did you find it easy or difficult in any way? Why?
20. Did you have any questions about the research (DMA/ TDF) during that time or maybe later? Did you find answers to your questions? Do you feel free to ask the researchers or doctors about issues or questions that might come up?
21. If you didn't feel able to ask your question, what needs to be done differently?

APPROVED
ETHICS COMMITTEE
FACULTY OF TROPICAL MEDICINE
MAHIDOL UNIVERSITY

<u>For spouse, family members or guardians</u>

1. Did the participant tell you about being asked to participate in the research (DMA/ TDF)? How did you react to it?
2. What do you think about her or his participation?
3. Why did you decide to let her participate?
4. Was it difficult to make the decision? Why or why not?
5. How does participating in research affect you and your family? Are there any benefits or any negative consequences?

Wrap-up

22. What was the general attitude (opinion?) towards the research in your community?
23. Do you have any ideas or suggestions for how the researchers can make participation easier for you and your family?
24. Are any extra precautions/ safety measures needed?
25. Speaking hypothetically, what other types of health-related research/service would you or your community like to see taking place?
26. Do you have any questions for us or anything else you would like to tell me?

-END-

APPROVED
ETHICS COMMITTEE
FACULTY OF TROPICAL MEDICINE
MAHIDOL UNIVERSITY
