# Supplementary material for: Culturally responsive research ethics: How the socio-ethical norms of Arr-nar/Kreng-jai inform research participation at the Thai-Myanmar border
Source: PLOS Glob Public Health. 2023 May 4;3(5):e0001875. doi: 10.1371/journal.pgph.0001875 (PMC10159138; doi:10.1371/journal.pgph.0001875)
Supplement: S2 Appendix — (PDF) [file pgph.0001875.s002.pdf]

Topic Guide for Interviews and Focus Group Discussion
Group 2 and 3

Challenges, problems, difficulties in daily life:

1. Could you tell me about the kinds of challenges/ problems facing people in this community?
   Probe: Health, food, education, livelihoods, housing, citizenship, access to services
   a. How do these problems affect people's lives?
   b. What causes these problems?

2. Are there any people or households that are more affected/ at risk than others? In what way?

Sources of support, mitigating factors, coping, resilience:

3. Are there things that people do to try to prevent/ protect themselves from any of the problems we were discussing? [Probe for each problem]

4. If they do experience these problems, what (if anything) do they/ their households do to cope? [Probe for each problem]
   a. Do they rely on anyone/anything for help? Who and in what way?
   b. How easy or difficult is it for people to access these kinds of support?

5. Are any people/ households less able to protect themselves from and/or cope with problems than others? Why and in what way?

Research ethics – how is research adding benefit, addressing vulnerability, or adding to burdens:

Research participants

6. Could you describe the groups of people who are usually asked to participate in research here?
   a. Are there any types people more likely to be excluded from research studies than others? Why?
   b. Anyone you think shouldn't be included? Why?
   c. Are there any people that you think should be included but often aren't? Why?

7. Are you aware of any ways that participating in research has been beneficial for people? [Probe for why/in what way]

8. Has participating in research created any difficulties for anyone? [Probe for why/in what way]
   a. Do some participants experience more difficulties than others? [Probe for why/in what way]

APPROVED
ETHICS COMMITTEE
FACULTY OF TROPICAL MEDICINE
MAHIDOL UNIVERSITY

Digitally signed by Faculty of Tropical Medicine, Mahidol University
DN: c=TH, st=Bangkok, l=Ratchathewi, o=Faculty of Tropical Medicine, Mahidol University, cn=Faculty of Tropical Medicine, Mahidol University, email=tmectropmed@mahidol.ac.th
Date: 2018.11.01 16:05:14 +07'00'

9. Do you have any suggestions for what researchers could do to make participation easier for participants?

Research and the community

10. Do any research studies create benefits for the community? [Probe for which/ in what way]

11. Do any of them cause problems or are burdensome for the community? [Probe for which/ in what way]

12. How does the community feel about the research studies that take place here? [Probe positive/ negative feelings and why]

13. Thinking about some of the specific problems we discussed earlier - do you think researchers/ research studies should have any responsibility for responding to these kinds of problems? In what way?
    a. How could they do this better?

For researchers/ healthcare workers

14. What ethical challenges have you faced carrying out research with women and children in this community?
    a. How have you responded to these challenges?
    b. What special precautions have you taken to protect 'vulnerable' participants?

For Tak Province Community Advisory Board (T-CAB) /Ethics Committee (EC) Members/ Clinical Trials Support Group (CTSG) members

15. First, what kinds of research take place in this community?
    a. Who is involved in deciding what research takes place? Who has final say?

16. What are the main things you check for / think about when you're reviewing research proposals?
    a. Is this at all different depending on the type of research/ who will be involved?

17. Are there any types of research you would/ have turned down? If yes, why? [Probe for examples]

18. What are the main challenges you experience as a CAB/EC/CTSG? As a member?

APPROVED
ETHICS COMMITTEE
FACULTY OF TROPICAL MEDICINE
MAHIDOL UNIVERSITY

Wrap-up

19. If you could, is there anything else you would change about the way the research studies are planned or carried out? How could they be improved?

20. We have covered a lot of territory – is there anything else you would like to add, or any other thoughts about this general topic you would like to share with me?

For researchers, study coordinators and study administrators only:

We know you are very busy so I really appreciate you taking time to offer your insights on this topic. In addition to speaking researchers, study coordinators and study administrators one-on-one and in group discussions, we wanted to ask you to keep a simple, private diary of ethical challenges that arise in your day-to-day work, and solutions or strategies for responding. This could be a small pocket diary provided by us or an electronic document.

Entries can be very short and simple, or longer if you have time. It is up to you.

Please make sure that you do not record names or identifying details within these entries. Information pocket diaries should be kept locked in a secure location, such as your desk in the clinic. Electronic diary entries should be password protected and saved on a secure server.

We will send a weekly reminder, prompting you to briefly reflect on the prior week and whether any issues or challenges arose. In about four months we will collect your diaries. Your name and identity will in no way be attached to these and we will take care to omit any accidental references to names or identifying details when these are transcribed.

Example entry:

Weekly Entry: Date:

1. Any ethical challenges that arose in your research work this week? Briefly describe the issue.

2. Strategies or solutions? How was it handled? If ongoing please follow up with update later.

-End-

APPROVED
ETHICS COMMITTEE
FACULTY OF TROPICAL MEDICINE
MAHIDOL UNIVERSITY
