# Supplementary material for: Culturally responsive research ethics: How the socio-ethical norms of Arr-nar/Kreng-jai inform research participation at the Thai-Myanmar border
Source: PLOS Glob Public Health. 2023 May 4;3(5):e0001875. doi: 10.1371/journal.pgph.0001875 (PMC10159138; doi:10.1371/journal.pgph.0001875)
Supplement: S1 File — (PDF) [file pgph.0001875.s003.pdf]

For manuscript title: Culturally responsive research ethics: How the socio-ethical norms of Arr-nar/Kreng-jai inform research participation at the Thai-Myanmar Border

Consolidated criteria for reporting qualitative studies (COREQ): 32-item checklist
Developed from:
Tong A, Sainsbury P, Craig J. Consolidated criteria for reporting qualitative research (COREQ): a 32-item checklist for interviews and focus groups. *International Journal for Quality in Health Care*. 2007. Volume 19, Number 6: pp. 349 – 357

Item no.	Topic	Guide questions/description	Response and/or location in manuscript (section, page no.)
**Domain 1: Research team and reflexivity**<br>*Personal Characteristics*			
1	Interviewer/facilitator	Which author/s conducted the interview or focus group?	Interviews and focus group discussions were led by NK, SN, NJ, and MK (Page 8)
2	Credentials	What were the researcher's credentials? E.g. PhD, MD	Authors with PhD include BH, MP, FN, RM, PYC, and MK. Authors with MD include RM, FN. SA and JR holds an M.Sc. JR is a DPhil candidate.
3	Occupation	What was their occupation at the time of the study?	All authors were researchers at their respective institutions.<br>In addition, BH, FN, RM, MP, PYC, and MK were also professors.
4	Gender	Was the researcher male or female?	Female researchers include NK, SN, BH, NJ, JR, RM, PYC, and MK<br><br>Male researchers include SA, MP, FN
5	Experience and training	What experience or training did the researcher have?	All researchers had experiences carrying out qualitative research. NK, SN have deep understandings of the research setting and the cultural context, and were trained to conduct interviews.<br>SA, NJ, JR, MK had training in social science research.
Relationship with participants			
6	Relationship established	Was a relationship established prior to study commencement?	No.
7	Participant knowledge of the interviewer	What did the participants know about the researcher? e.g. personal goals, reasons for doing the research	Name, and reasons for carrying out research.
8	Interviewer characteristics	What characteristics were reported about the inter viewer/facilitator? e.g. Bias, assumptions, reasons and interests in the research topic	Name, and reasons for carrying out research.
**Domain 2: study design**<br>*Theoretical framework*			
9	Methodological orientation and Theory	What methodological orientation was stated to	This was an embedded empirical ethics case study, including ethical analysis and

		underpin the study? e.g. grounded theory, discourse analysis, ethnography, phenomenology, content analysis	thematic discourse analysis of qualitative data in research ethics in ethnographic context. The analysis was informed by a grounded understanding of the ethical concepts of Arr-nar and Kreng-jai and core concepts of international research ethics. (Section: Data analysis, pages 10 to 13)
Participant selection			
10	Sampling	How were participants selected? e.g. purposive, convenience, consecutive, snowball	Participants were selected through purposive sampling within a case study design. Some expert stakeholders were identified through snowball recruitment.<br><br>(Section: Study design, recruitment and data collection, page 5-7)
11	Method of approach	How were participants approached? e.g. face-to-face, telephone, mail, email	Face-to-face;<br>Telephone;<br>Email invitation
12	Sample size	How many participants were in the study?	32 in-depth interviews, and<br>10 focus group discussions<br>(in total 48 persons)<br><br>(Table 2, page 8)
13	Non-participation	How many people refused to participate or dropped out? Reasons?	There were no refusals.
Setting			
14	Setting of data collection	Where was the data collected? e.g. home, clinic, workplace	The data were collected at the SMRU's network of clinics along the Thai-Myanmar border near Mae Sot, Thailand. A few interviews were carried out at participants' preferred location (home or office).<br><br>FGDs were carried out in the conference room at the SMRU's main offices in Mae Sot, Thailand.
15	Presence of non-participants	Was anyone else present besides the participants and researchers?	Most interviews were held in a private space/room.<br><br>For those few interviews held in a participants' home by request, participant's family member(s) were in nearby room. For some interviews with women, their infant/child was present.<br><br>The focus groups were held in a closed conference room with only participants and facilitators present for the duration.
16	Description of sample	What are the important characteristics of the sample? e.g. demographic data, date	Participants included migrant women participating in one of two clinical studies being conducted at SMRU; expert and community stakeholders familiar

			with the Thai-Myanmar border context; and clinical researchers and clinical staff from SMRU. Participant groups and demographics are described in detail in Tables 1 pages 6-7 and Table 2 page 8.<br><br>Data collection was conducted from December 2017 to March 2019.
Data collection			
17	Interview guide	Were questions, prompts, guides provided by the authors? Was it pilot tested?	Yes. Included in Supporting Information.<br><br>Yes, we practiced with the guide to check for flow and timing.
18	Repeat interviews	Were repeat interviews carried out? If yes, how many?	Yes, there were 3 follow-up interviews.
19	Audio/visual recording	Did the research use audio or visual recording to collect the data?	Yes, audio recording was used. (Page 9)
20	Field notes	Were field notes made during and/or after the interview or focus group?	During and after.
21	Duration	What was the duration of the interviews or focus group?	IDI: 30 minutes to 60 minutes<br>FGD: 45 minutes to 90 minutes
22	Data saturation	Was data saturation discussed?	Yes. (Page 8.)
23	Transcripts returned	Were transcripts returned to participants for comment and/or correction?	No, transcripts were not returned.
**Domain 3: analysis and findings**<br>*Data analysis*			
24	Number of data coders	How many data coders coded the data?	Four (NK, SA, SN, and NJ) with periodic discussions with JR, PYC, and MK.<br><br>(Page 10-11.)
25	Description of the coding tree	Did authors provide a description of the coding tree?	No.
26	Derivation of themes	Were themes identified in advance or derived from the data?	Themes were derived from the data.
27	Software	What software, if applicable, was used to manage the data?	NVivo 12
28	Participant checking	Did participants provide feedback on the findings?	We did not perform formal participant checking due to the difficulty of tracing migrant participants. However, de-identified aggregate findings were presented and discussed with a subset of participants prior to publications. These presentations/workshop included

			participants from the research and staff group, and community stakeholders who serve on the Community Advisory Board.
Reporting			
29	Quotations presented	Were participant quotations presented to illustrate the themes/findings? Was each quotation identified? e.g. participant number	Yes. / Yes.<br><br>(Result section, pages 13-26.)
30	Data and findings consistent	Was there consistency between the data presented and the findings?	Yes.
31	Clarity of major themes	Were major themes clearly presented in the findings?	Yes.
32	Clarity of minor themes	Is there a description of diverse cases or discussion of minor themes?	Yes, we include and describe 'sub-themes' within the broader major ethical themes from the data. We also describe ethical/cultural ambiguity where relevant to appreciating the theme or subtheme in cultural context. We are careful to note when views were expressed did not reflect the majority of participants, but were nonetheless important for appreciating the range of ethical views.
