# Supplementary material for: Culturally responsive research ethics: How the socio-ethical norms of Arr-nar/Kreng-jai inform research participation at the Thai-Myanmar border
Source: PLOS Glob Public Health. 2023 May 4;3(5):e0001875. doi: 10.1371/journal.pgph.0001875 (PMC10159138; doi:10.1371/journal.pgph.0001875)
Supplement: S2 File — Abstract in Burmese language. (PDF) [file pgph.0001875.s004.pdf]

ထုတ်နှုတ်ချက် - ယဉ်ကျေးမှုအလိုက် တုံ့ပြန်ဆောင်ရွက်နိုင်စွမ်းရှိသော သုတေသနကျင့်ဝတ်များအ တွက် သင်ခန်းစားများ : ထိုင်း-မြန်မာ နယ်စပ်ရှိ သုတေသနပါဝင်လုပ်ဆောင်မှုတွင် 'အားနာခြင်း/ခရန် ဂျိုင်' ဟူသည့် လူမှု-ကျင့်ဝတ်ဆိုင်ရာ စံများကို မည်သို့ သိရှိထည့်သွင်းလုပ်ဆောင်ထားသည်။

တိုးတက်မှုများရှိသော်လည်း နိုင်ငံတကာ သုတေသနကျင့်ဝတ်လမ်းညွှန်ချက်များသည် အနောက်အမေရိကန်နှင့် ဥရောပ ကျင့်ဝတ်ထုံးတမ်းများမှ လက်ကျန်လွှမ်းမိုးမှုများ ထင်ဟပ်လျက်ရှိသည့် အဆင့်မြင့်ကျင့်ဝတ်အခြေခံမှုများပါဝင်လေ့ရှိသည်။ ဒေသန္တရ ကျင့်ဝတ်ကော်မတီများနှင့် ရပ်ရွာလူထုအကြံပေးဘုတ်အဖွဲ့များသည် လေ့ကျင့်သင်ကြားပေးရာတွင် ယဉ်ကျေးမှုအရထိရှလွယ်မှုကို သိရှိလုပ်ဆောင် သည့် ချဉ်းကပ်နည်းများပိုမိုပေးအပ်နိုင်သော်လည်း အသင်းအဖွဲ့/ဌာနအများစုတို့သည် စုံလင်ကွဲပြား သောယဉ်ကျေးမှုနောက်ခံအနေအထားများအတွင်း နေ့စဉ် သုတေသနလုပ်ဆောင်မှုများအတွင်း ကျွယ် ဝသည့် အကျင့်စာရိတ္တပိုင်းဆိုင်ရာနားလည်သဘောပေါက်မှုများဖြင့် စေ့စပ်လုပ်ဆောင်ရန် အထင်ကရ လက်တွေ့ကျသည့် ကျင့်ဝတ်လမ်းညွှန်ချက်များ မရှိနေပါ။ ယင်းလစ်လပ်မှုကို ဖြေရှင်းရန်အတွက် ကျွန်ုပ်တို့သည် စုံလင်ကွဲပြားသော အနေအထားများအတွင်း အသက်ဝင်နေသည့် သုတေသနစီမံကိန်း များ တစ်လျှောက် ချိတ်ဆက်ပြီး၊ ကျင့်ဝတ်ဆိုင်ရာ ဖြစ်စဉ်လေ့လာမှုများအား အရည်အသွေးအခြေပြု သုတေသနတစ်ရပ်ကို နိုင်ငံတကာလိုက်လေ့လာမှုအဆင့်ဆင့် လုပ်ဆောင်ခဲ့သည်။ ဤစာတမ်းတွင် ထိုင်း-မြန်မာ နယ်စပ်တစ်လျှောက် ရွှေ့ပြောင်းသမားများအတွက် ဝန်ဆောင်မှုပေးနေသည့် ဆေးခန်းများ ရှိ ကိုယ်ဝန်ဆောင်အမျိုးသမီးများအတွက် ငှက်ဖျားရောဂါနှင့် အသည်းရောင်အသားဝါ ဘီပိုး ရောဂါကာ ကွယ်ရေးဆိုင်ရာ အကြောင်းအရာများအပေါ် သုတေသနလုပ်ကိုင်နေသည့် အဖွဲ့နှင့် ဖြစ်စဉ်ရပ်လေ့လာ ချက် (case study) တို့မှ တွေ့ရှိချက်များကို ကျွန်ုပ်တို့မျှဝေပေးထားပါသည်။ ဤ လူမှုယဉ်ကျေးမှုကျင့်ဝတ်ပိုင်းဆိုင်ရာ စိတ်ဖြာသုံးသပ်ချက်တွင် ကျွန်ုပ်တို့သည် စိတ်ဆန္ဒသဘောအရ ပါဝင်လုပ်ဆောင်ခြင်း၊ မျှတသော အကျိုးကျေးဇူးများပေးအပ်ခြင်းနှင့် သုတေသနအန္တရာယ်များအားနားလည်သဘောပေါက်ခြင်းနှင့် လိုက်နာရမည့်တာဝန် စသည့် အဓိက ကျင့်ဝတ်ပိုင်းဆိုင်ရာ လိုအပ်ချက်များအား အားနာခြင်း (ဗမာနှင့် ကရင် ဘာသာဖြင့်) သို့မဟုတ် ခရန်ဂျိုင် (ထိုင်းဘာသာဖြင့်) ဟုလူသိများသည့်၊ အချိန်ကာလကြာမြင့်စွာတည်ရှိထွန်းကားနေခဲ့ပြီးဖြစ်သည့် ဗမာ၊ ကရင်၊ နှင့် ထိုင်း ယဉ်ကျေးမှု စံနှုန်းများက မည်သို့ ပုံဖော်ထားသည်၊ အားကောင်းစေသည်၊ နှင့် အချို့ဖြစ်စဉ်များတွင် စိန်ခေါ်မှုရှိစေသည်တို့ကို - အခြားသူများ နှင့် ပူးပေါင်းဖော်ရွေမှု အတွက် စဉ်းစားချက်များ အပါအဝင် အမျိုးမျိုးသော အဓိပ္ပာယ်များကို ခြုံငုံထည့် သွင်းပြီး - ကျွန်ုပ်တို့ စဉ်းစားသုံးသပ်ပြထားသည်။ ကျွန်ုပ်တို့အနေဖြင့် သုတေသနလုပ်ဆောင်ချက် လမ်းကြောင်းတစ်လျှောက် ကျင့်ဝတ်ပိုင်းဆိုင်ရာအရထင်ရှားသည့် လူမှုယဉ်ကျေးမှုဆိုင်ရာလွှမ်းမိုးမှုများအား မည်သို့ဖော်ထုတ်နတိုင်သည့်အကြောင်း စံပုံစံ (model) ကို လည်း ရေးဆွဲပြသထားပြီးနောက်၊ အခြား နိုင်ငံတကာ အနေအထားများတွင် ယဉ်ကျေးမှုပိုင်းအရ တုံ့ပြန်ဆောင်ရွက်မှုပိုမိုအားကောင်းသော သု တေသန ကျင့်ဝတ် ကျင့်သုံးမှုအလေ့အထများ ဖော်ဆောင်ရန် သင်ခန်းစာများဖြင့် နိဂုံးချုပ်ထားသည်။
